# Sterol-Response Pathways Mediate Alkaline Survival in Diverse Fungi

**DOI:** 10.1128/mBio.00719-20

**Published:** 2020-06-16

**Authors:** Hannah E. Brown, Calla L. Telzrow, Joseph W. Saelens, Larissa Fernandes, J. Andrew Alspaugh

**Affiliations:** a Departments of Molecular Genetics and Microbiology, Duke University School of Medicine, Durham, North Carolina, USA; b Department of Medicine, Duke University School of Medicine, Durham, North Carolina, USA; c Faculty of Ceilândia, University of Brasília, Brasília, Federal District, Brazil; University of Toronto

**Keywords:** *Cryptococcus neoformans*, ergosterol, fungal genetics, membrane, pH homeostasis

## Abstract

The ability for cells to maintain homeostasis in the presence of extracellular stress is essential for their survival. Stress adaptations are especially important for microbial pathogens to respond to rapidly changing conditions, such as those encountered during the transition from the environment to the infected host. Many fungal pathogens have acquired the ability to quickly adapt to changes in extracellular pH to promote their survival in the various microenvironments encountered during a host infection. For example, the fungus-specific Rim/Pal alkaline response pathway has been well characterized in many fungal pathogens, including Cryptococcus neoformans. However, alternative mechanisms for sensing and responding to host pH have yet to be extensively studied. Recent observations from a genetic screen suggest that the C. neoformans sterol homeostasis pathway is required for growth at elevated pH. This work explores interactions among mechanisms of membrane homeostasis, alkaline pH tolerance, and Rim pathway activation. We find that the sterol homeostasis pathway is necessary for growth in an alkaline environment and that an elevated pH is sufficient to induce Sre1 activation. This pH-mediated activation of the Sre1 transcription factor is linked to the biosynthesis of ergosterol but is not dependent on Rim pathway signaling, suggesting that these two pathways are responding to alkaline pH independently. Furthermore, we discover that C. neoformans is more susceptible to membrane-targeting antifungals under alkaline conditions, highlighting the impact of microenvironmental pH on the treatment of invasive fungal infections. Together, these findings further connect membrane integrity and composition with the fungal pH response and pathogenesis.

## Abstract (precis)

The work described here further elucidates how microorganisms sense and adapt to changes in their environment to establish infections in the human host. Specifically, we uncover a novel mechanism by which an opportunistic human fungal pathogen, Cryptococcus neoformans, responds to increases in extracellular pH in order to survive and thrive within the relatively alkaline environment of the human lung. This mechanism, which is intimately linked with fungal membrane sterol homeostasis, is independent of the previously well-studied alkaline response Rim pathway. Furthermore, this ergosterol-dependent alkaline pH response is present in Candida albicans, indicating that this mechanism spans diverse fungal species. These results are also relevant for novel antimicrobial drug development as we show that currently used ergosterol-targeting antifungals are more active in alkaline environments.

## INTRODUCTION

Diverse cell types, from simple unicellular microorganisms to complex multicellular eukaryotes, interpret alterations in extracellular pH as a common signal for changes in the external environment. Pathogenic microorganisms are often uniquely exposed to wide fluctuations in pH as they move between various microenvironments in the human host. Among these, fungi that cause invasive fungal infections (IFIs) have acquired the ability to rapidly adapt to changes in extracellular pH to promote their survival during an infection. The shift of a fungal pathogen from an acidic external environment to the neutral/alkaline pH of the mammalian host is associated with the activation of the fungus-specific Rim/Pal signaling pathway, triggering cellular changes important for survival under these new conditions. These changes include alterations in the cell wall, often accompanied by larger morphological transitions that promote host colonization. In the common fungal pathogen Candida albicans, pH-directed cellular responses include the ability to transition between yeast-like growth and invasive hyphal forms (1–3). The opportunistic human fungal pathogen and basidiomycete yeast Cryptococcus neoformans similarly activates Rim signaling to respond to changes in pH. Because C. neoformans initially colonizes the human lung, which is often relatively more alkaline than its natural environmental reservoirs, this signaling pathway is activated in the setting of infection. In fact, the C. neoformans Rim101 transcription factor, the terminal component of the Rim pathway, is among the most highly induced transcripts *in vivo* (4).

Given its pH-dependent activation, as well as its important role in the adaptation of fungal cells to elevated pH, the Rim signaling cascade is often considered to be the major alkaline pH response pathway in fungi. However, other cellular processes and pathways are required for fungal growth under conditions of extreme pH (both acidic and alkaline). These processes include the production of glycosphingolipids (GSLs) that associate with proteins in the outer leaflet of fungal plasma membranes to form lipid rafts and maintain membrane fluidity and organization (5–7). Recent studies have demonstrated that mutations resulting in reduced or absent GSLs render fungi such as Kluyveromyces lactis, Neurospora crassa, and C. neoformans unable to grow in alkaline environments (8–11). The connection between membrane composition and the ability for fungal cells to grow in alkaline environments has been associated with defects in cytokinesis and altered activity of plasma membrane proton pumps, as well as an altered lipid profile (10). Furthermore, reduced ergosterol content in membranes has been linked to salt stress sensitivity in Saccharomyces cerevisiae (12, 13) and to aberrant V-ATPase regulation of pH gradients in Candida albicans (13, 14).

Recent observations from our genetic screen suggest that C. neoformans sterol homeostasis might also be required for growth at elevated pH (15). The sterol homeostasis pathway (SREBP pathway) has been extensively studied in both mammalian and fungal cells (16–20). Proteins in this pathway regulate the production and delivery of sterols to the plasma membrane to maintain appropriate cell homeostasis (17, 21, 22). In several fungal species, including C. neoformans, the Sre1 transcription factor (the terminal transcription factor in this sterol homeostasis pathway) is activated in response to low-oxygen conditions (21, 23–26). In addition to hypoxia, the C. neoformans Sre1 transcriptional response is necessary for tolerance to low iron and to antifungals that target sterols in the membrane (21). Upon activation of the C. neoformans sterol homeostasis pathway, the basidiomycete-specific Stp1 protease cleaves Sre1, freeing its N terminus to release from the membrane of the endoplasmic reticulum and translocate to the nucleus (22). This cleavage is induced in an O_2_-dependent manner and is important for the transcription of many ergosterol biosynthesis genes (23, 25). However, the association between the sterol homeostasis pathway and pH adaptation has not yet been explored.

Here, we define potential interactions among fungal sterol homeostasis, alkaline pH tolerance, and Rim pathway activation. We find that the sterol homeostasis pathway is indeed necessary for growth in an alkaline environment and that an elevated pH is sufficient to induce Sre1 cleavage and activation. This pH-mediated activation of the Sre1 transcription factor is not dependent on Rim pathway signaling, suggesting that these two pathways are responding to alkaline pH independently. Furthermore, we demonstrate that Sre1-mediated ergosterol biosynthesis is linked to the response to alkaline pH and relevant in biologically diverse fungi. Finally, we discover that C. neoformans is more susceptible to membrane-targeting antifungals under alkaline conditions, highlighting the impact of microenvironmental pH on the treatment of this invasive fungal infection. Together, these findings connect a highly conserved pathway involved in membrane homeostasis and sterol maintenance to the adaptive response to changes in extracellular pH.

## RESULTS

### Convergent and divergent phenotypes of the *sre1*Δ and *rim101*Δ mutants.

**FIG 1:**
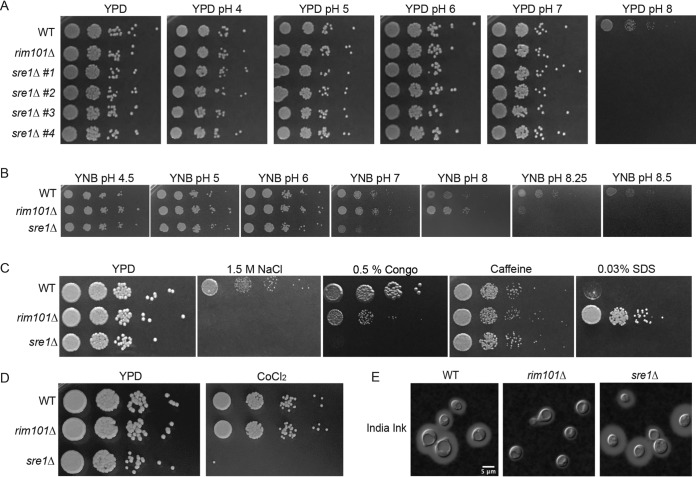
Stress response phenotypes of the *sre1*Δ and *rim101*Δ mutant strains. (A) Four independent *sre1*Δ mutant strains were serially diluted onto YPD medium and YPD pH 4 to 8. Growth was compared to wild type (WT) and a *rim101*Δ mutant known to have alkaline pH sensitivity. Growth was assessed after 3 days. *sre1*Δ #1 (HEB5) is shown for all subsequent phenotyping and analysis. (B) The *sre1*Δ and *rim101*Δ mutant strains are unable to grow at increasing pH levels on minimal medium (YNB). Strains were spotted in serial dilutions onto YNB medium buffered to pH 4.5 to 8.5, and growth was compared to WT after 3 days. (C) The *sre1*Δ and *rim101*Δ mutant strains display distinct and overlapping phenotypes to cell stressors. Strains were serially diluted and spotted to either YPD, YPD plus 1.5 M NaCl, YPD plus 0.5% Congo red, YPD plus 1 mg/ml caffeine, or YPD plus 0.03% SDS. Growth was compared to WT and assessed after 3 days. (D) The *sre1*Δ mutant strain displays a growth defect in response to hypoxia-mimicking growth conditions (7 mM CoCl_2_). Strains were spotted in serial dilutions onto YPD at 30°C and YES plus 7 mM CoCl_2_ at 30°C. Growth was assessed after 3 days and compared to WT and the *rim101*Δ mutant. (E) The *sre1*Δ mutant strain does not have the same capsule deficiency as the *rim101*Δ mutant strain. Strains were incubated in CO_2_-independent medium for 3 days before imaging using India ink exclusion counterstaining. Capsule is noted as a halo of clearing around the yeast cells.

A recent forward genetic screen identified two elements of the C. neoformans sterol homeostasis pathway, the Sre1 transcription factor and its activating protease Stp1, as proteins required for growth of this pathogenic fungus at an alkaline pH (15). To confirm this observation, we generated and acquired multiple, independent C. neoformans *sre1*Δ mutants and verified that all demonstrated a severe growth defect at high pH (Fig. 1A). We performed detailed phenotypic comparisons between mutants in the alkaline-responsive Rim pathway and the sterol homeostasis pathway, as exemplified by the *rim101*Δ and *sre1*Δ transcription factor mutant strains, respectively. Both mutant strains grew similarly to wild type (WT) on a rich growth medium at pH 5.5 (yeast extract-peptone-dextrose [YPD] medium). These two mutants also displayed similar growth defects as the wild type on growth medium buffered to a pH greater than 7 (Fig. 1A). Importantly, the *sre1*Δ alkaline pH-sensitive mutant phenotype was rescued by the reintroduction of the wild-type *SRE1* allele (see Fig. S1A in the supplemental material).

10.1128/mBio.00719-20.1 FIG S1 The *sre1*Δ mutant phenotypes are rescued by the reintroduction of the wild-type *SRE1* allele, demonstrating a distinct growth defect from the *rim101*Δ mutant under microaerophilic conditions. (A) Strains were spotted in serial dilutions onto YPD pH 5.5, YPD pH 8, and YES plus 7 mM CoCl_2_. Growth was assessed after 3 days of growth and compared to both the *sre1*Δ mutant and WT strains. (B) The *sre1*Δ and *stp1*Δ mutant strains displayed an expected growth rate and colony size reduction in response to microaerophilic growth conditions. The *rim101*Δ mutant was able to grow in this environment similarly to wild-type levels. Strains were spotted in serial dilutions onto YPD at 30°C and YPD at 30°C and incubated under microaerophilic conditions. Download FIG S1, TIF file, 1.0 MB. Copyright © 2020 Brown et al. 2020 Brown et al. This content is distributed under the terms of the Creative Commons Attribution 4.0 International license.

To account for a potential confounding effect on growth by exogenous lipids in the yeast extract-rich medium, we also assessed the ability for these mutant strains to grow on a minimal medium (yeast nitrogen base [YNB]) buffered to a range of pH values. The *sre1*Δ and *rim101*Δ mutants were able to grow on YNB medium buffered to pH 4 through pH 7. At more alkaline pH, the growth defect of the *sre1Δ* mutant strain was more severe than that of the *rim101*Δ mutant, with the *sre1*Δ mutant unable to grow at pH 8 and the *rim101*Δ strain displaying complete growth inhibition only at pH >8 (Fig. 1B).

Given the established role of Sre1 in mediating growth in hypoxia, we compared growth rates of these mutant strains in the presence of cobalt chloride (CoCl_2_), an agent that disrupts many biochemical pathways, including the ergosterol biosynthesis pathway and cellular respiration (27–30). Consistent with previous reports (21, 24, 25, 31), the *sre1*Δ mutant is unable to grow under these conditions (Fig. 1D and Fig. S1A). The *rim101*Δ mutant did not share this growth defect and was not sensitive to CoCl_2_ (Fig. 1D). We also compared growth rates of all mutant strains in a microaerophilic chamber to more directly test phenotypes in response to reduced oxygen. The sterol homeostasis pathway mutants displayed a lower growth rate under conditions of reduced oxygen concentration (Fig. S1B). The *rim101*Δ mutant grew to similar levels as the wild type (Fig. S1B). Therefore, although sharing a similar alkaline growth defect, the *rim101*Δ and *sre1*Δ mutants display distinct growth patterns under hypoxia-like conditions.

We also tested the sensitivity of the *sre1*Δ and *rim101*Δ mutant strains to cell wall stressors such as Congo red (interferes with beta glucan-chitin linkages), caffeine (affects cell wall integrity), high salt (osmotically stresses the cell wall), and SDS (stresses the cell membrane) (32, 33). Similarly to alkaline pH, high salt resulted in complete growth inhibition for both mutant strains (Fig. 1C). In contrast, caffeine did not affect the growth of either mutant (Fig. 1C). The *sre1*Δ mutant strain was unable to grow in the presence of Congo red, whereas the *rim101*Δ mutant strain showed only a subtle growth defect due to this chitin polymer inhibitor (Fig. 1C). Also, SDS completely inhibited growth of the *sre1*Δ strain, whereas the *rim101*Δ strain appeared to be hyperresistant to the membrane-targeting effects of SDS, as evident in the more robust growth of this strain than of the wild type (32, 34) (Fig. 1C).

Sensitivities of mutant strains to cell surface stressors can indicate alterations in the cell wall structure and/or integrity. In addition to providing a protective barrier for the cell, the cell wall serves as an anchor for the attachment of the polysaccharide capsule that can further protect the fungal cells during a human infection (35). The *rim101*Δ mutant strain is known to have a disorganized cell wall and thus a decrease in attached capsule (36, 37). In contrast, the *sre1*Δ mutant strain revealed intact capsule formation (38) (Fig. 1E). Overall, these phenotypic comparisons distinguish the *rim101*Δ mutant from the *sre1*Δ mutant in the distinct responses of these strains to cell wall and membrane stress.

### Independent signaling of the Rim and sterol homeostasis pathways.

**FIG 2:**
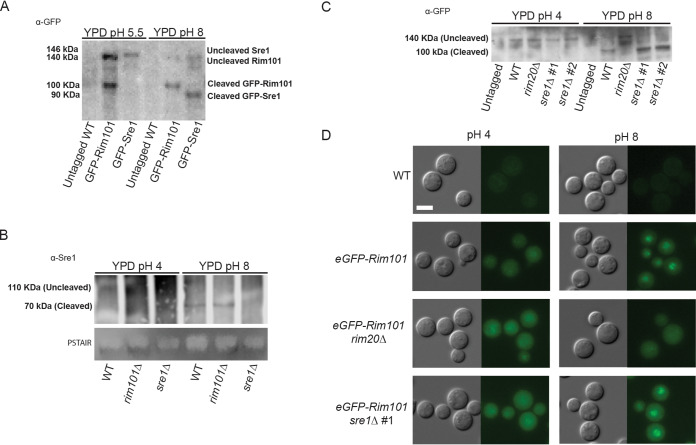
Sre1 activation is dependent on alkaline pH but not Rim signaling. (A) Western blot of both Sre1 and GFP-Rim101 protein processing under low-pH and high-pH growth conditions. The GFP-Rim101 fusion protein is cleaved from its 140-kDa form to its active 100-kDa form at pH 8. Similarly, the GFP-Sre1 fusion protein is proteolytically processed from 146 kDa to approximately 90 kDa in response to alkaline pH. Indicated strains were incubated for 60 min in either pH 5.5 or pH 8 YPD medium prior to lysing. Rim101 and Sre1 protein processing was determined using a GFP-trap pulldown and Western blotting using an anti-GFP antibody. Protein levels were normalized prior to loading. (B) Western blot analysis of the Sre1 protein in different genetic backgrounds revealed the cleavage and processing (from 110 kDa to approximately 90 kDa) of the Sre1 transcription factor in the WT and *rim101*Δ mutant backgrounds. Indicated strains were incubated for 60 min in either pH 4 or pH 8 YPD medium prior to lysing. Protein processing was determined through protein A pulldown and Western blotting using a polyclonal anti-Sre1 antibody. Total protein levels are represented by a PSTAIR loading control. (C) The Sre1 protein is cleaved in response to alkaline pH. The *eGFP-RIM101* allele was expressed in the WT, *rim20*Δ mutant, and two independent *sre1*Δ mutant strains (*sre1*Δ #1 and *sre1*Δ #2). The untagged WT strain and the eGFP-Rim101-expressing strains were incubated in YPD medium pH 4 or pH 8 for 60 min. Rim101 processing was assessed using a GFP-trap pulldown and Western blotting using an anti-GFP antibody. Protein levels were normalized prior to loading. (D) The indicated strains (the same as in panel C) were incubated in synthetic complete medium buffered to pH 4 or pH 8 for 60 min. Rim101 localization was assessed by epifluorescence microscopy, and alkaline-induced nuclear localization was compared to the eGFP-Rim101 positive control. White scale bars indicate 5 μm.

To determine whether the C. neoformans sterol homeostasis pathway is specifically activated in response to alkaline pH, we assessed the pH dependence of the proteolytic cleavage of the Sre1 transcription factor, a marker of pathway activation (21, 23–25, 38). At pH 5.5, the green fluorescent protein (GFP)-Sre1 fusion protein remains uncleaved in a 140-kDa form (Fig. 2A). In contrast, incubating this strain in the same growth medium buffered to pH 8 results in GFP-Sre1 protein cleavage to a 90-kDa form, similar to its proteolytic activation in response to hypoxia (Fig. 2A) (21). There was no defect in Sre1 cleavage in the *rim101Δ* mutant strain background (Fig. 2B). Therefore, the C. neoformans sterol homeostasis pathway is specifically activated by an alkaline pH signal and in a manner that is independent of the Rim alkaline response pathway.

To further define the interaction between the Sre1 and Rim101 signaling pathways, we assessed whether the Sre1 transcription factor is necessary for activation of the Rim pathway as measured by the pH-dependent proteolytic processing and subcellular localization of the Rim101 transcription factor (39). In both the wild-type and *sre1*Δ mutant strains, we observed intact Rim101 processing and cleavage at elevated pH (Fig. 2C). Similarly, GFP-Rim101 nuclear localization was enhanced at activating pH in both strain backgrounds (Fig. 2D). In contrast, we confirmed both defective protein cleavage and impaired nuclear localization of the Rim101 transcription factor in the *rim20*Δ mutant, a strain lacking a known upstream Rim signaling component (Fig. 2C and D). These data indicate that the sterol homeostasis pathway is not required for Rim pathway activation.

### The cell wall organization of the *sre1*Δ mutant and its *in vitro* immune phenotypes.

**FIG 3:**
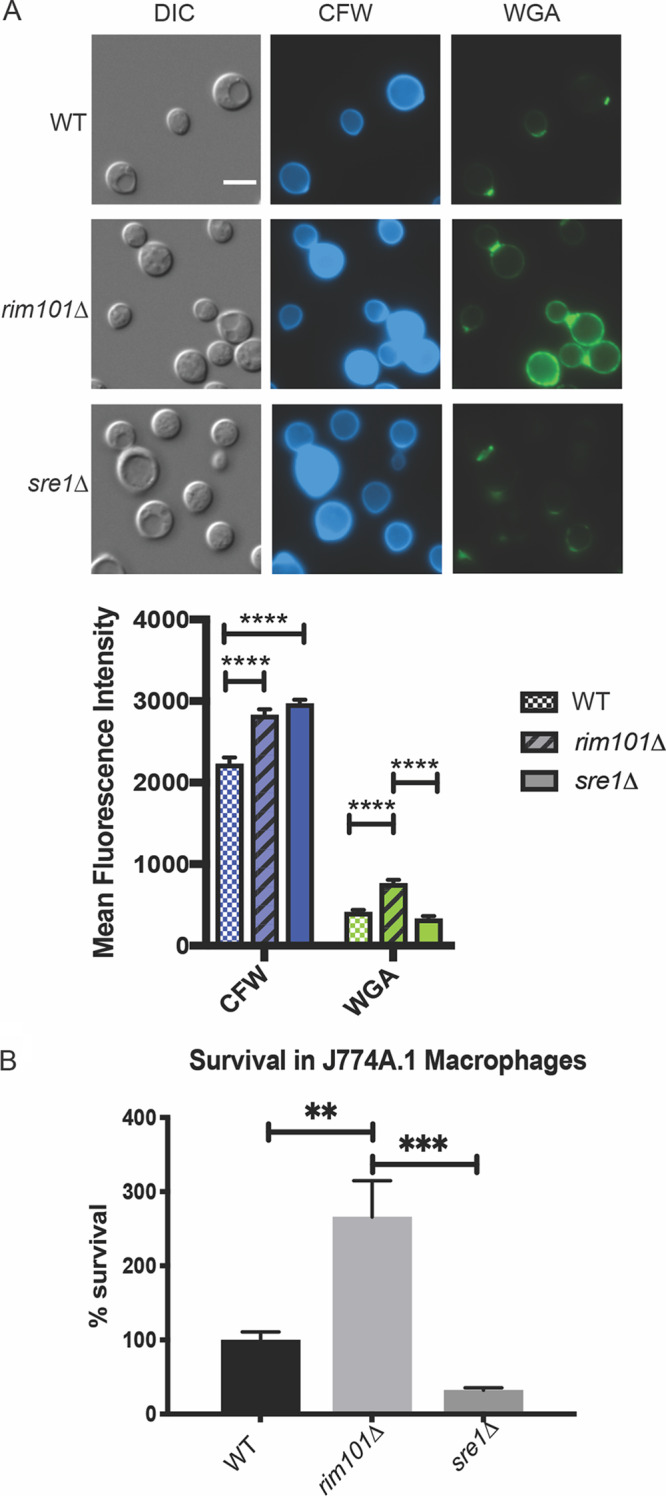
*sre1*Δ and *rim101*Δ mutant strains have varied changes in cell wall chitin exposure and interactions with host immune cells. (A) Staining of *rim101*Δ, *sre1*Δ, and wild-type cells with calcofluor white (CFW) and wheat germ agglutinin (WGA). Cells were incubated in CO_2_-independent medium for 18 h at 37°C. Cells were stained with FITC-conjugated WGA and CFW and incubated in the dark for 35 min and 10 min, respectively. Mean fluorescence intensity was quantified for each strain and each condition. Two-way ANOVA and Tukey’s multiple-comparison test were run to determine statistical significance. White scale bars indicate 5 μm. ****, *P* value < 0.0001. DIC, differential inference contrast. (B) When grown in the presence of J774A.1 macrophages, the *rim101*Δ mutant strain can survive significantly better than both the wild-type and the *sre1*Δ mutant strain. Indicated strains were coincubated with macrophages for 24 h, and survival was determined by quantitative cultures. One-way ANOVA and Tukey’s multiple-comparison tests were run to assess statistical significance between fungal cell survival percentages. Six biological replicates of each strain were analyzed. **, *P* value < 0.003; ***, *P* value < 0.0002.

The *sre1*Δ mutant strain is avirulent in a mouse model of C. neoformans infection (25, 38), whereas the *rim101*Δ mutant strain and other Rim pathway mutants have paradoxical hypervirulent phenotypes in the same model (36). In previous work, we demonstrated by transmission electron microscopy that the *rim101*Δ mutant has an aberrant, thick, and disorganized cell wall in comparison to wild-type cells (36). We probed the *rim101*Δ and *sre1*Δ mutant strains with calcofluor white (CFW) and wheat germ agglutinin (WGA) to assess total and exposed levels of chitin, respectively. In both mutant strains, we noted similar increases in cell wall chitin levels as measured by CFW staining. However, the level of exposed chitin (WGA) was increased only in the *rim101Δ* strain. The intensity of the WGA fluorescence was quantified by measuring brightness intensity (Fiji) in photomicrographs (Fig. 3A) as well as by flow cytometry (Fig. S2). The observed increase in total chitin levels can be a nonspecific response to cell stress (40). However, increased chitin exposure, as assessed by intensity of WGA staining, has been previously demonstrated to correlate with the degree of macrophage activation *in vitro* (36, 41). Together, these cell wall analyses suggest that the Rim pathway and sterol homeostasis pathway induce distinct microbial physiological responses to host-like conditions. Specifically, the Sre1-mediated response to host stress does not include increased exposure of chitin.

10.1128/mBio.00719-20.2 FIG S2 Quantitative analysis of exposed chitin levels in *rim101*Δ and *sre1*Δ mutant cell walls. The *rim101*Δ cell wall has increased exposed chitin staining by flow cytometry compared to WT and the *sre1*Δ mutant cell walls. (A and C) All strains were incubated for 16 to 18 h at 30°C in YPD medium (A) or 37°C in TC medium (C), fixed, labeled, and analyzed by flow cytometry. Wheat germ agglutinin (WGA) was used to stain the cell walls for exposed chitin. (A and C) Positive events were gated in the forward scatter (FSC)/side scatter (SSC) plots and are represented as histograms with cell counts on the *y* axis and mean fluorescence on the *x* axis. Unstained cells were sorted as controls to determine positive events. (B and D) The geometric means from positive events for each strain. Download FIG S2, TIF file, 1.0 MB. Copyright © 2020 Brown et al. 2020 Brown et al. This content is distributed under the terms of the Creative Commons Attribution 4.0 International license.

To further define the extent to which these cell wall epitopes may affect virulence, we assessed macrophage interactions with the *sre1*Δ mutant compared to the *rim101*Δ mutant strain. Macrophages are among the first immune cells encountered by this pathogen when infecting its host in the human lung. We therefore quantified fungal survival after coculturing stimulated J774A.1 murine macrophage-like cells with the wild-type, *rim101*Δ, and *sre1*Δ strains. Following coincubation with macrophages, the *rim101*Δ mutant strain displayed increased survival compared to wild type, as has been shown previously (37) (Fig. 3B). The *sre1*Δ mutant strain displayed a moderate, reproducible reduction in viability in the presence of macrophages compared to the wild-type strain. This result was consistent with the previously reported attenuated virulence of the *sre1*Δ mutant strain in animal models of infection (25, 38). The significantly different patterns of macrophage interaction of the *sre1*Δ and *rim101*Δ mutant strains further suggest that distinct downstream cellular processes are controlled by these alkaline-responsive pathways (Fig. 3B).

### Ergosterol biosynthesis is required for growth at alkaline pH in C. neoformans and other fungal pathogens.

**FIG 4:**
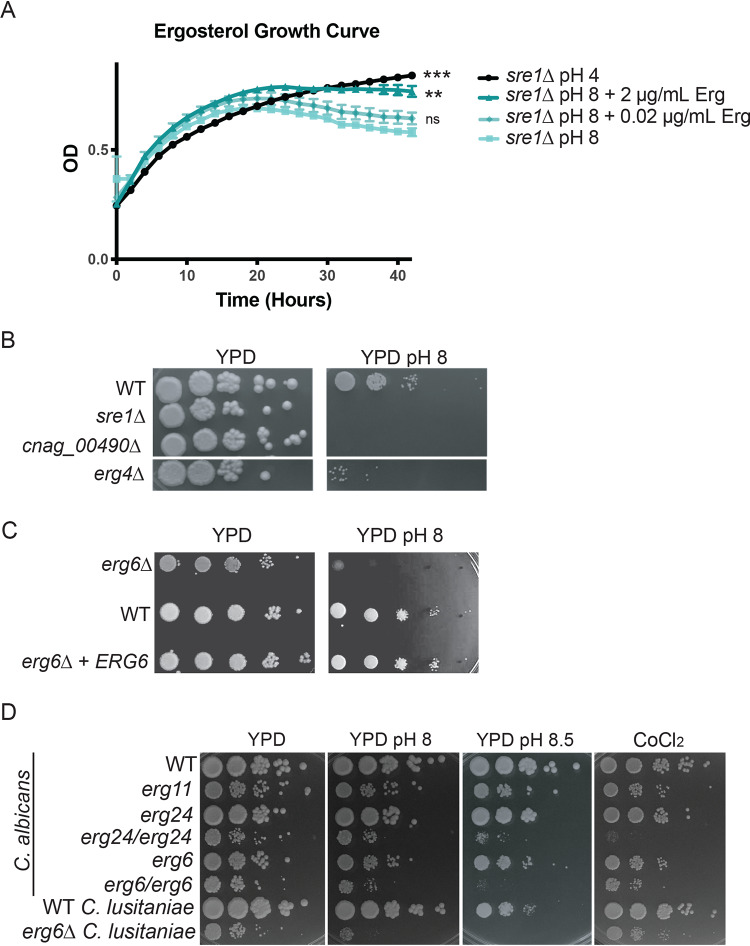
Altered ergosterol content renders strains sensitive to alkaline pH. (A) The reduced growth rate of the *sre1*Δ mutant strain in liquid growth medium at pH 8 can be rescued through the addition of exogenous ergosterol in a dose-dependent manner. Growth rate of indicated strains was assessed by changes in OD_595_ in biological triplicate every 10 min for 42 h at 30°C. Ergosterol was added as indicated. One-way ANOVA and Dunnett’s multiple-comparison test were run on the last time point under each condition compared to the pH 8-alone condition to determine statistical significance. **, *P* value < 0.003; ***, *P* value < 0.0005; ns, not significant. (B) Other sterol-related mutants exhibit alkaline pH sensitivity. Two deletion mutants related to ergosterol biosynthesis in C. neoformans (*erg4Δ* and *cnag_00490*Δ) display a pH sensitivity when grown on pH 8 growth medium. Indicated strains were serially diluted onto YPD medium and YPD-150 mM HEPES pH 8. Growth was compared to WT and assessed after 3 days. (C) *erg6*Δ C. neoformans mutant also exhibits alkaline pH sensitivity when grown on pH 8 medium. Indicated strains were serially diluted onto YPD medium and YPD-150 mM HEPES pH 8. Growth was compared to WT and reconstituted strains and assessed after 3 days. (D) *Candida* species ergosterol mutants reveal similar pH-sensitive phenotypes. C. albicans and C. lusitaniae wild-type strains and strains with mutations in various components of ergosterol biosynthesis were serially diluted onto YPD medium and YPD-150 mM HEPES pH 8 and 8.5 as well as YES medium with 7 mM CoCl_2_. Growth was compared to WT and assessed after 2 days.

Our data support that the Rim and sterol homeostasis pathways are independent cell signaling pathways that each mediate adaptive responses to alkaline stress. Given the established role of fungal Sre1 orthologs in the regulation of membrane sterol content, we hypothesized that alterations in minor membrane lipids, especially ergosterol, might be involved in the adaptive response to alkaline pH. Previous work in C. neoformans sterol homeostasis documented decreased ergosterol levels in the *sre1*Δ mutant strain (22, 25). The *sre1*Δ alkaline pH sensitivity was rescued by the addition of exogenous ergosterol to the growth medium in a dose-dependent manner (Fig. 4A). Importantly, addition of exogenous sterols did not affect the pH of the growth medium. This observation is similar to prior investigations showing growth rescue of various S. cerevisiae ergosterol biosynthesis mutants by supplementation with exogenous ergosterol (42). These data suggest that intact ergosterol induction and homeostasis are specifically required for fungal adaptation to alkaline pH.

To further explore the role of ergosterol biosynthesis in the alkaline pH response, we tested three C. neoformans ergosterol-related mutants for growth at pH 8, and all shared an alkaline pH growth defect (Fig. 4B). Many steps in ergosterol biosynthesis are essential for growth under routine conditions, limiting the availability of *ERG* gene mutants. The nonessential *ERG4* and *ERG6* genes encode terminal enzymes in the ergosterol biosynthesis pathway (22, 43). Compared to wild type, the *erg4*Δ and *erg6*Δ mutants displayed a specific growth defect at alkaline pH (Fig. 4B and C) (15). Similarly, the *CNAG_00490* locus encodes a putative acetyl coenzyme A (acetyl-CoA) acetyltransferase, as does the *ERG10* (*CNAG_02918*) gene. The loss-of-function *cnag_00490Δ* mutant also displays alkaline pH sensitivity (Fig. 4B). The pH sensitivity of the *CNAG_00490* mutant as well as the predictive function of its gene product suggests that it might participate in the conversion of acetyl-CoA to squalene, an early step in sterol synthesis.

Ergosterol is a major component of most fungal membranes, including those of distantly related fungal pathogens in the ascomycete phylum. To further explore the association between sterol homeostasis and alkaline pH response, we tested alkaline pH survival for ergosterol biosynthesis mutants in two *Candida* species, C. albicans and C. lusitaniae (Fig. 4D). The homozygous diploid C. albicans *erg6*/*erg6* and *erg24/erg24* mutants displayed severe growth defects at high pH that were not evident under more acidic conditions (Fig. 4D). Similarly, the haploid C. lusitaniae *erg6* mutant had impaired growth compared to wild type under alkaline conditions (Fig. 4D). These results suggest a conserved requirement for efficient sterol maintenance in the adaptation to alkaline pH among highly divergent fungal species.

### Sre1 regulates membrane-associated transcripts under alkaline growth conditions.

**FIG 5:**
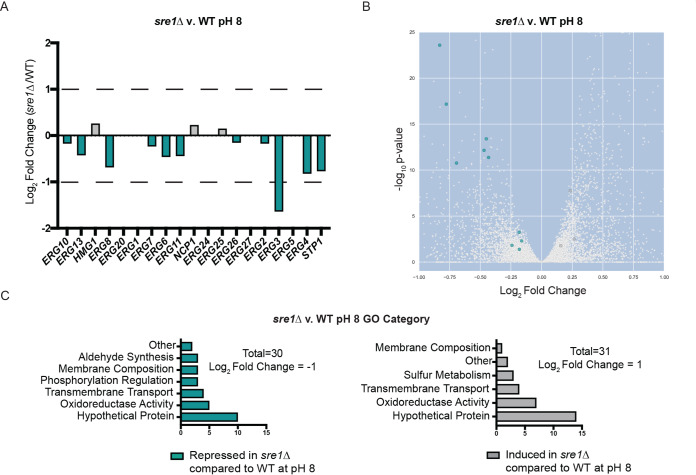
Transcriptomic analysis of the *sre1*Δ and wild-type strains in response to alkaline pH. WT and *sre1*Δ cells were incubated in YPD medium pH 4 or pH 8 for 90 min. This experiment was conducted with six biological replicates for each strain and condition. Total RNA was extracted, mRNA was isolated, and libraries were prepared and finally sequenced using an Illumina NextSeq 500 sequencer. GO-term analysis was performed using FungiDB. (A) The majority of the known genes in C. neoformans ergosterol biosynthesis were significantly differentially expressed in the *sre1*Δ versus wild-type transcriptome at pH 8. *ERG* genes that were significantly differentially expressed have an adjusted *P* value of <0.016 (teal, repressed in the *sre1*Δ mutant compared to wild type; gray, induced in the *sre1*Δ mutant compared to wild type). (B) Volcano plot displaying the significantly regulated transcripts in the *sre1*Δ versus wild-type transcriptome at pH 8 (adjusted *P* value of <0.05) (teal, repressed in the *sre1*Δ mutant compared to wild type; gray, induced in the *sre1*Δ mutant compared to wild type). The full volcano plot (zoomed out) is shown in Fig. S2. (C) GO-term analysis of the *sre1*Δ versus wild-type differentially expressed genes following a 90-min shift from YPD pH 4 to YPD pH 8. These transcripts were selected based on a strict cutoff of log_2_ fold change of ±1. Teal, biological processes repressed in *sre1*Δ mutant compared to wild type at high pH; gray, biological processes induced in *sre1*Δ mutant compared to wild type.

The C. neoformans *SRE1*-dependent transcriptome has been defined in the context of the cellular response to low oxygen (21, 22, 38). These prior studies revealed that Sre1 is required for the induction of genes involved in ergosterol homeostasis in an oxygen-dependent manner. However, given the novel role for Sre1 pathway activation at alkaline pH, we defined the pH-responsive Sre1-regulated transcriptional response. Comparison of the transcriptomes of the *sre1*Δ mutant and wild type after 1.5 h of growth in alkaline pH revealed 2,655 transcripts that were differentially regulated in a statistically significant manner (adjusted *P* value of <0.05) (Fig. S3A and Table S1). This represents approximately one-quarter of the C. neoformans genome, indicating that Sre1 has a major impact on the cell in response to pH stress. Similar to the transcriptome studies in hypoxia, transcript abundance of the majority of the *ERG* genes (13/18) and the *STP1* activating protease was differentially regulated at alkaline pH (Fig. 5A and B). The *stp1*Δ mutant strain displays a pH-sensitive mutant phenotype similar to the *sre1*Δ mutant strain (15). Importantly, *ERG3* transcript levels had the highest relative fold change in the *sre1*Δ mutant at high pH compared to wild type (Fig. 5A and B). *ERG3* encodes a component of the ergosterol biosynthesis pathway and displays similar Sre1-dependent expression under low-oxygen conditions (22).

10.1128/mBio.00719-20.3 FIG S3 Full transcriptome analyses and WT GO terms. (A) Volcano plot of the full transcriptome as analyzed from the data set for *sre1*Δ mutant versus WT at pH 8 (cropped version in Fig. 5B). (B) Volcano plot of the wild-type transcriptome. (C and D) GO-term analysis of the wild-type differentially expressed genes that are induced (C) and repressed (D) following a 90-min shift from YPD pH 4 to YPD pH 8. These transcripts were selected based on a strict cutoff of log_2_ fold change of +1 for induced transcripts and log_2_ fold change of −3 for repressed transcripts based on the uneven distribution of total repressed transcripts shown on the volcano plot (B). Download FIG S3, TIF file, 0.3 MB. Copyright © 2020 Brown et al. 2020 Brown et al. This content is distributed under the terms of the Creative Commons Attribution 4.0 International license.

10.1128/mBio.00719-20.5 TABLE S1 RNA-seq analysis. Page 1. *sre1*Δ versus wild-type differentially expressed genes at pH 8 (repressed). Annotation of GO terms and putative processes/functions included. *P* < 0.05 and log_2_ fold change < −1. Page 2. *sre1*Δ versus wild-type differentially expressed genes at pH 8 (induced). Annotation of GO terms and putative processes/functions included. *P* < 0.05 and log_2_ fold change < 1. Page 3. *sre1*Δ versus wild-type entire transcriptome at pH 8. Raw data. Page 4. Wild-type pH 4 versus wild-type pH 8 entire transcriptome. Raw data. Page 5. Wild-type pH 4 versus wild-type pH 8 differentially expressed genes (induced). Annotation of GO terms and putative processes/functions included. *P* < 0.05 and log_2_ fold change < 1. Page 6. Wild-type pH 4 versus wild-type pH 8 differentially expressed genes (repressed). Annotation of GO terms and putative processes/functions included. *P* < 0.05 and log_2_ fold change < −3. Download Table S1, XLSX file, 1.2 MB. Copyright © 2020 Brown et al. 2020 Brown et al. This content is distributed under the terms of the Creative Commons Attribution 4.0 International license.

Due to the large number of differentially expressed transcripts identified in this analysis, we performed a modified Gene Ontology (GO)-term analysis using FungiDB on genes with a 2-fold or greater change in transcript abundance in the *sre1*Δ mutant compared to wild type (44). Genes repressed in the *sre1*Δ mutant at high pH are enriched for biological processes such as aldehyde synthesis, cellular respiration/oxidoreduction, membrane composition, phosphorylation regulation, and transmembrane transport. Genes that are induced in this mutant background under alkaline conditions are involved in cellular respiration/oxidoreduction, membrane composition, sulfur metabolism, and transmembrane transport (Fig. 5C and Table S1). Interestingly, although some of these GO terms are shared with the previously published *SRE1* transcriptome under 3% oxygen conditions, the majority of the Sre1-dependent transcripts differ between the two experimental inducing conditions: hypoxia versus alkaline pH (22) (Fig. S4). Using the same fold change values to compare these transcript data sets, only nine genes are induced under both conditions, the majority of which are related to ergosterol biosynthesis: *SRE1*, *ERG3*, *ERG11*, *ERG6*, *ERG4*, and *ERG13* (Fig. S4). This transcriptome analysis supports the central role for ergosterol biosynthesis genes as potential Sre1-dependent effectors of both hypoxia and the response to alkaline pH. We also documented that different inducing conditions mediate distinct Sre1-dependent transcriptional responses.

10.1128/mBio.00719-20.4 FIG S4 Compared transcriptomics. Venn diagram showing the overlap between three different transcriptome data sets in C. neoformans serotype A (H99). Bien et al. (22) performed a comparative transcriptomics microarray study using the *sre1*Δ mutant strain and the *sre1*Δ + *SRE1* complemented strain in low-oxygen growth environments (3% O_2_) (dark blue). A 1.4-fold-change cutoff was applied to this data set, and the transcripts included in this comparison were increased in the reconstituted strain compared to the mutant. Our data set looking at the *sre1*Δ mutant compared to wild type at low pH (4) and high pH (8) is shown in teal. We originally applied a fold change cutoff of 2 to our data set to analyze our GO-term categories (Fig. 5C and D) but lowered the cutoff to 1.4 to match the work of Bien (2009) and included only those transcripts that were repressed in the *sre1*Δ mutant compared to wild type. The overlap of our data with the previously published data set revealed many ergosterol-related transcripts: *SRE1, ERG3*, *ERG4*, *ERG11*, *ERG6*, and *ERG13*. Download FIG S4, TIF file, 0.7 MB. Copyright © 2020 Brown et al. 2020 Brown et al. This content is distributed under the terms of the Creative Commons Attribution 4.0 International license.

We were also able to define groups of genes in the wild-type strain that are either induced or repressed following the shift from low to high pH. These groups include a significant portion of membrane-associated transcripts, including integral membrane components, composition regulators, and membrane transporters (Fig. S3C and D and Table S1). Transcripts with increased abundance in response to alkaline pH include many of the known Rim pathway regulators (*RIM101* and *RIM23*) and pathway outputs (*ENA1*, *CIG1*, and *SKN1*) (Fig. S3C and Table S1). Consistent with prior reports of the involvement of Sre1 in iron homeostasis (21, 45), we also identified an iron transporter (CNAG_00815), suggesting a conserved role for iron regulation to adapt to changes in extracellular pH (Table S1). Furthermore, many genes involved in membrane composition, glucose/complex carbohydrate metabolism, and regulation of protein phosphorylation were induced under alkaline conditions (Fig. S3C). Complex carbohydrates are major components of the fungal cell wall, supporting previous findings that the Rim-mediated pH response is linked to the reorganization of the cell wall (36). GO-term analysis of transcripts with reduced abundance at high pH revealed genes involved in membrane transport, potentially in an effort to regulate import of extracellular ions into the cell (Fig. S3D). This analysis revealed no clear repression of membrane composition transcripts at high pH (Fig. S3D and Table S1).

### pH affects efficacy of membrane-targeting antifungals.

**FIG 6:**
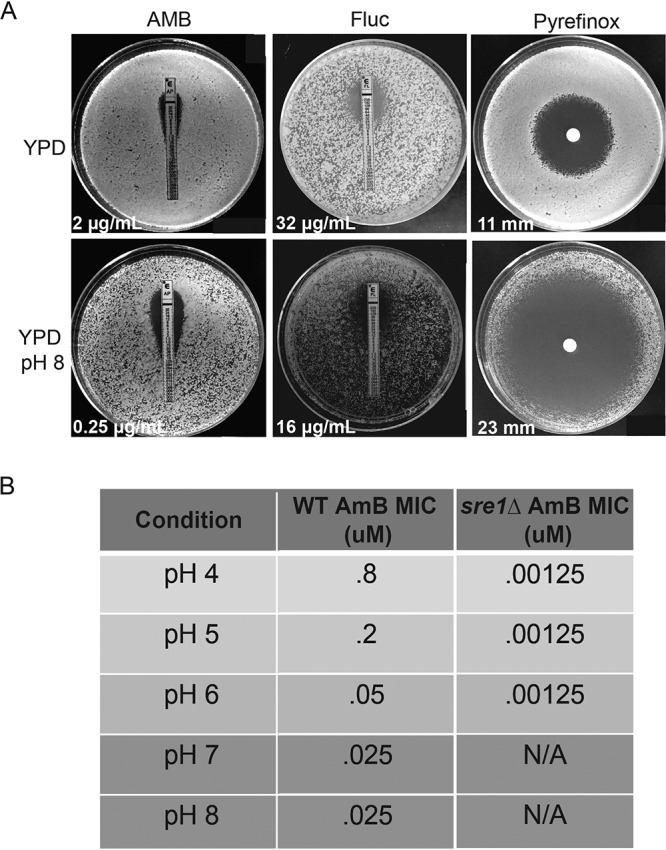
Membrane-targeting antifungals are more active at alkaline pH. (A) Assessing MICs and the zones of inhibition (white values) of membrane-targeting drugs (amphotericin B [AMB], fluconazole [Fluc], and pyrifenox) on wild-type cells grown on YPD or alkaline (YPD pH 8) medium. Measurements were taken after 5 days of growth for AMB and pyrifenox and 3 days of growth for Fluc. All plates were incubated at 30°C. (B) MIC of AMB for wild-type and the *sre1*Δ mutant C. neoformans strains grown under increasingly alkaline conditions. MIC was determined after 48 h of growth at 30°C by broth microdilution. MIC values could not be determined (N/A) for *sre1*Δ mutant at pH >6 due to the inability of this strain to grow under these more alkaline conditions.

Given our observation of a correlation between fungal sterols and growth at alkaline pH, we tested the pH-dependent efficacy of antifungal agents targeting different aspects of membrane ergosterol homeostasis. Amphotericin B (AMB) is a polyene antifungal that removes ergosterol from fungal membranes (46). We observed a dramatic reduction in the AMB MIC for wild-type C. neoformans cells grown on YPD pH 8 (0.25 μg/ml) compared to YPD pH 5.5 (2 μg/ml) (Fig. 6A). Furthermore, the time-dependent killing of fungal cells by AMB increased in a pH-dependent manner, further supporting that this drug has a higher efficacy under alkaline growth conditions (Fig. 6B). We also found that AMB was significantly more efficacious against the *sre1*Δ strain (MIC = 0.00125 μM) than the wild type when the cells were grown at low pH (pH 4 to 6) (Fig. 6B). The significant increase in AMB activity against this mutant strain with reduced ergosterol content is consistent with our model that disruption in fungal sterols leads to pH sensitivity. Furthermore, in a drug disc diffusion assay using pyrifenox, a drug used to treat phytopathogens through inhibition of ergosterol biosynthesis (47), there was a significantly greater zone of clearance and inhibition of growth of wild-type C. neoformans cells when grown on medium buffered to pH 8 compared to pH 5.5 (Fig. 6A).

Fluconazole is an antifungal that inhibits the activity of Erg11, an important component of the ergosterol biosynthesis pathway. We hypothesized that removing ergosterol from the cell membrane in this way would cause a similar sensitivity to alkaline pH as we observed with the ergosterol mutant strains in various fungal pathogens (Fig. 4). In contrast to the major pH-dependent activity of AMB and pyrifenox, we observed a reproducible but more subtle effect of pH on fluconazole efficacy. The fluconazole MIC was 2-fold lower for wild type at pH 8 (16 μM) compared to YPD pH 5.5 (32 μM) (Fig. 6A). The azoles and polyenes have been shown in other organisms, such as Aspergillus fumigatus, to have variable activity against invasive fungal infections depending on the pH of the growth environment (48). Similar to the findings in A. fumigatus, these data support that increases in alkalinity allow for higher efficacy of specific polyenes and azoles against C. neoformans. Our data reveal that reduction of ergosterol, either genetically or biochemically using known antifungals, leads to reduced growth in alkaline environments. Altogether, these results further inform the connection between fungal plasma membrane homeostasis, the molecular interactions that drive environment sensing, and the ability for a biologically diverse group of fungi to grow in increasingly alkaline environments, including their human host.

## DISCUSSION

### Novel, Rim-independent pH-sensing pathway in C. neoformans.

**FIG 7:**
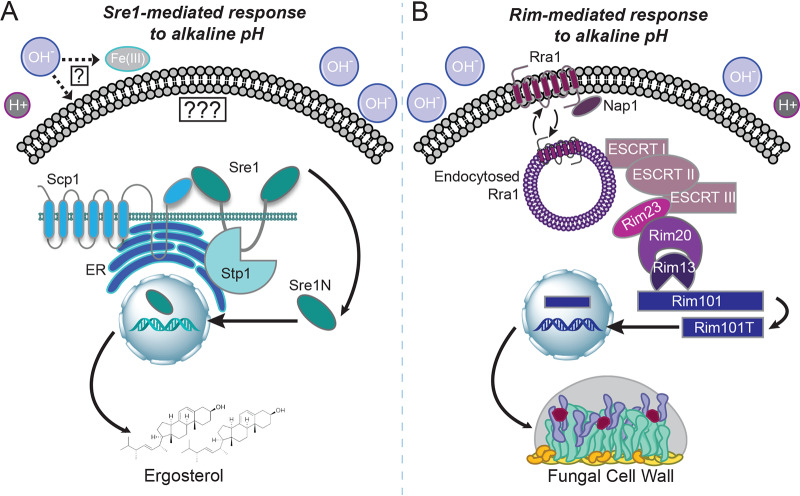
Model of the Sre1-mediated and Rim-mediated distinct responses to physiological pH. (A) The activating sensor for the sterol homeostasis pathway is unknown and could be linked to alkaline pH-induced reductions in ergosterol/membrane alterations or bioavailable iron. In response to alkaline pH, the Sre1 transcription factor is cleaved, activated, and localized to the nucleus to aid in the transcription of many genes involved in ergosterol biosynthesis and membrane homeostasis. This cleavage and activation are dependent on both the conserved transmembrane protein Scp1 and the basidiomycete-specific protease Stp1. (B) The Rim alkaline response pathway is signaled through the transmembrane pH sensor Rra1 and its interaction with the plasma membrane. At elevated pH levels, Rra1 is endocytosed, allowing it to interact with the downstream components of the pathway and propagate the signal to the endosomal membrane complex (ESCRT components, Rim23, and Rim20) and activate the Rim13 protease. This protease cleaves the Rim101 transcription factor, allowing it to translocate to the nucleus and induce the expression of genes involved in cell wall and surface remodeling.

These experiments support a model in which several cell processes and signaling pathways work together to allow microbial growth under stress conditions such as elevated pH. The Rim signaling pathway has been identified in multiple fungal species including C. neoformans, C. albicans, and S. cerevisiae as a major signaling response to increases in extracellular pH (1–3, 15, 36, 37, 39, 49–53) (Fig. 7). Its primary function appears to be translating extracellular alkaline pH signals to control adaptive changes in the fungal cell wall (36) (Fig. 7B). Data presented in this study identified the sterol homeostasis pathway as a unique mechanism that responds to alkaline pH in a Rim-independent way (Fig. 7).

The sterol homeostasis pathway has been implicated in the response to alterations in oxygen availability, membrane ergosterol levels, and various stressors in diverse fungal species. In the fission yeast, Schizosaccharomyces pombe, the induction of ergosterol biosynthesis genes by the Sre1 transcription factor and its chaperone proteins (Scp1 and Ins1) has been well characterized in response to hypoxia (18, 20, 54). C. neoformans, similarly to S. pombe, has a well characterized Sre1-mediated response to hypoxia that results in the induction of ergosterol biosynthesis genes to maintain membrane homeostasis (21, 25, 27, 34, 38). However, in C. neoformans, a basidiomycete-specific protease has been identified that specifically activates Sre1 in response to hypoxia (Fig. 7A) (22, 38). Elements of this pathway have also been identified in the filamentous fungal pathogen A. fumigatus. The Sre1 homolog, SrbA, is essential for the ability of this pathogen to grow in environments with limited oxygen or low iron or in the presence of membrane-targeting antifungals (19, 23, 45, 55, 56). This hypoxic response is required for survival in the infected host in which hypoxic microenvironments exist, especially in poorly viable tissue such as necrotic tumors and wounds (57). The dimorphic fungal pathogen Histoplasma capsulatum also contains a homolog of Sre1 (Srb1) that is essential for the response to hypoxia as well as for virulence (58–60). Other yeasts such as C. albicans and S. cerevisiae do not contain genes in their sequenced genomes encoding obvious SREBP homologs. Instead, these species respond to hypoxic stress through the activation of a different transcription factor, Upc2, which directs the induction of ergosterol biosynthesis genes (61, 62). However, the C. albicans Cph2 protein binds SRE1-like elements in the genome, and it may therefore be a functional ortholog of Sre1 (63).

The identification of a new role for the sterol homeostasis pathway is informative to better conceptualize and target fungal pathogenesis in general and cryptococcal pathogenesis in particular for several reasons. First, the sterol pathway in C. neoformans has a basidiomycete-specific Stp1 protease that is required for cleavage and activation of Sre1 (22, 25, 38). Genes encoding a similar protease are found in the genomes of other basidiomycete fungi such as Cryptococcus gattii, Malassezia globosa, and Mucor circinelloides (44) and not in those of more distantly related fungi or higher eukaryotes. This fungal specificity and distinction from the mammalian sterol homeostasis pathway (16, 17, 64) may provide an interesting future target for novel antifungals. Second, understanding the extracellular cues that activate this pathway may elucidate more detailed signaling mechanisms controlling sterol homeostasis, potentially revealing some currently unknown upstream components. Presently, it is not known if a common signal in hypoxia or alkaline pH initiates Sre1 signaling, or if multiple upstream Sre1 activators are present (Fig. 7A). The C. neoformans sterol homeostasis pathway is lacking an obvious INSIG homolog as well as a site-1 protease (24). Elucidating the Sre1-mediated response to alkaline pH through further analysis of our forward genetic screen may uncover either functional orthologs of these proteins or novel pathway components that mediate specific stress responses in C. neoformans.

The transcriptional analysis of the *sre1*Δ mutant strain at high pH provided further support for the distinct activation of the Sre1 transcription factor in response to increases in extracellular pH. This type of analysis has been conducted for the C. neoformans *sre1*Δ mutant strain previously but with conditions of low and high oxygen availability (22). When comparing our transcriptomics data to this previously published microarray analysis, the majority of the transcripts were nonoverlapping, suggesting independent downstream effectors of Sre1 in response to specific stress (see Fig. S4 in the supplemental material). Furthermore, there was no overlap between the Sre1-associated transcriptome at high pH and the previously published Rim101-associated transcriptome at a similar pH, further supporting the distinct nature of these two pH response mechanisms and the specificity of the Sre1-mediated response to alkaline pH stress (data not shown and Fig. 7) (15).

### Ergosterol biosynthesis is essential for the ability of fungal pathogens to grow in an alkaline environment.

The generation of ergosterol for overall fungal membrane integrity has been well studied in the response to extracellular stresses such as hypoxia and low iron (23, 24, 26, 38, 45, 55, 56). Ergosterol controls the fluidity and structure of fungal cells (65), and it is needed for the formation of microdomains within the membrane containing ion pumps and transmembrane proteins necessary for cellular growth and signaling (12, 13, 65–67). In this study, we have demonstrated that supplementing pH-sensitive mutant strains with ergosterol can rescue the pH-sensitive mutant phenotype, suggesting that the *sre1*Δ mutant pH sensitivity is specifically linked to its ergosterol deficiency.

Our studies further supported this link between ergosterol and the pH response through analysis of the effects of alkaline pH on the biosynthesis of ergosterol at the transcriptional level. In response to a shift in pH, the majority of the known C. neoformans ergosterol biosynthesis genes were differentially regulated in the *sre1*Δ strain compared to wild type. These results support our model and implicate Sre1-mediated membrane homeostasis as a direct response to alkaline stress (Fig. 7A). Furthermore, C. neoformans and C. albicans strains with mutations in known and predicted ergosterol synthetic processes were unable to grow at alkaline pH. These results indicate that ergosterol levels and membrane homeostasis are important in the pH response mechanisms of many fungal species. This broadens these findings from Sre1-specific regulation of ergosterol affecting pH growth of a basidiomycete fungal pathogen to general ergosterol maintenance affecting the pH response in many different fungal pathogens across phyla.

In addition to establishing a link between alkaline pH and membrane sterols, our data also support emerging data on the interplay between the pH of the external environment and iron homeostasis (Fig. 7A). In divergent cell types, bioavailable iron concentrations are often reduced at alkaline pH (68). Our data demonstrate the induction of an iron transporter transcript in response to alkaline pH (Table S1), further suggesting that the cell is responding to reduced iron availability under this condition. In A. fumigatus, supplementing *ΔsrbA* mutants with exogenous iron rescues growth defects in low oxygen and during azole treatment (45). Also, in the dimorphic fungal pathogen H. capsulatum, Sre1 signaling mediates the ability of this fungus to survive under hypoxic conditions as well as to control iron regulation. Each of these processes may mediate separate roles in fungal virulence (59, 60) Furthermore, prior investigations have also demonstrated that the C. neoformans Sre1-mediated stress response is linked to iron availability (21). Future studies will determine if exogenous iron will fully or partially suppress the C. neoformans *sre1*Δ mutant pH growth defects in a similar manner as exogenous sterols.

### Ergosterol-depleting antifungals render cryptococcal cells sensitive to alkaline pH.

Our results have shown not only that genetic manipulation of fungal membrane homeostasis and ergosterol biosynthesis can increase the sensitivity of C. neoformans to alkaline pH but also that biochemical and pharmaceutical interventions have the same effect. We tested relevant antifungals that prevent sterol production or directly deplete sterols from fungal membranes and demonstrated that the activity of these drugs improves in neutral/alkaline environments. AMB, an antifungal that directly disrupts the plasma membrane through sequestration of ergosterol (46), was significantly more potent with increases in the pH of the growth environment. Similarly, fluconazole and pyrifenox, drugs that inhibit the ergosterol biosynthesis pathway (47, 69), were also more effective at alkaline pH. These results reflect similar findings in *Aspergillus* species treated with itraconazole and AMB (48). Similar studies using ketoconazole, AMB, and flucytosine (5-FC) against *Candida* species showed that the *in vitro* drug activity increases as a function of pH (70, 71). Interestingly, there has also been one study demonstrating increased efficacy of 5-FC against C. neoformans at higher pH (72). The fact that flucytosine does not directly target the cell membrane, together with the subtle alterations in fluconazole activity as a function of pH, suggests that multiple factors control this phenomenon. However, our findings that known ergosterol-targeting antifungals render diverse fungi more vulnerable to growth environments with increasing pH further support our leading hypothesis that ergosterol homeostasis is a central contributor to the alkaline pH response of many fungal pathogens.

Translating basic investigations in the role of pH modulation in human disease into potential clinical applications has precedent in cancer biology. In mammalian cells, studies of pH regulation in tumor metastasis demonstrated an association between the pH within a tumor and the degree of tumor cell apoptosis, survival, and proliferation (73). The preference among certain malignant cells for more acidic external environments has prompted the exploration of “buffer therapy,” in which site-directed pH modulation is used as an adjunctive therapy to limit tumor growth (74). This type of therapy is also effective against microbial infections that colonize the airways and intestines, such as Pseudomonas aeruginosa and Escherichia coli, respectively (75–77). If these interventions can be used against bacterial infections, one might imagine how similar pH modulation could be specifically applied to combat the acidic, necrotic core of many established invasive fungal infections, including cryptococcal lesions (57, 78, 79). Understanding pH-mediated microbial changes in various host microniches will allow for the development of optimized antifungal activity at the site of infection.

## MATERIALS AND METHODS

### Strains, media, and growth conditions.

Strains generated and/or utilized in this study are shown in Table 1. Each mutant, reconstituted strain, and fluorescent strain were generated in the C. neoformans H99 *MAT*α genetic background and incubated in either yeast-peptone-dextrose medium (YPD) (1% yeast extract, 2% peptone, and 2% dextrose) or yeast nitrogen base medium (YNB). The pH 4, 5, 5.5, 6, 7, and 8 media were made by adding 150 mM HEPES buffer to YPD or YNB medium, adjusting the pH with concentrated HCl (for pH <5.5) or NaOH (for pH >5.5), prior to autoclaving. Medium was supplemented with 20% glucose following autoclaving unless otherwise noted. Cell wall stress phenotypes were assessed by growth on various stress medium agar plates as previously described (32). Congo red (0.5%) and NaCl (1.5 M) were added to YPD medium prior to autoclaving. Caffeine (1 mg/ml) and SDS (0.03%) were filter sterilized and added to YPD medium following autoclaving. Cobalt chloride plates were made by adding 7 mM (90.89 mg/liter) CoCl_2_ solution to autoclaved YES medium (glucose, yeast extract, adenine, uracil, histidine, leucine, lysine, and agar) (80, 81). Capsule induction and analyses were completed as previously described (32). Briefly, strains were incubated overnight in YPD medium and then diluted in tissue culture medium (CO_2_-independent tissue culture medium [TC]; Gibco) for 72 h with shaking at 37°C and then counterstained with India ink. The microaerophilic conditions were generated using a sealed chamber (BD GasPak) and two activated packs of GasPak EZ Campy container system (containing campylobacter) to reduce oxygen levels. YPD plates with serial dilutions of normally grown strains were placed in the chamber for 24 h at 37°C (microaerophilic) or outside the chamber for 24 h at 37°C (ambient air).

**TABLE 1:** Strain list

Strain	Genotype	Reference or source
H99	*MAT*α	95
TOC35	*rim101Δ*::*NAT*	37
HEB5	*sre1*::*NEO MAT*α (#1)	15
HEB6	*sre1*::*NEO MAT*α (#2)	This study
YSB2493	*sre1*::*NAT MAT*α (#3)	34
YSB2494	*sre1*::*NAT MAT*α (#4)	34
HEB94	*sre1*::*NEO* + *His-SRE1*(*NAT*) *MAT*α	This study
HEB71	*His-GFP-Sre1 MAT*α	This study
KS91	*His-GFP-Rim101 MAT*α	96
TOC106	*eGFP-Rim101 MAT*α	96
HEB13	*eGFP-Rim101* + *sre1*::*NEO MAT*α 1	This study
HEB14	*eGFP-Rim101* + *sre1*::*NEO MAT*α 2	This study
KS118-2	*rim20*::*NAT eGFP-Rim101 MAT*α	39
KS33	*rim13*::*NEO MAT*α	39
HM.5-F6^*a*^	*erg4Δ*::*NAT MAT*α	97
HM.21-E12^*a*^	*cnag_00490Δ*::*NAT MAT*α	97
*erg6Δ*	*erg6*::*HPH* (hygromycin resistance)	43
SC5314	WT Candida albicans	98
4A	*erg11/ERG11* Candida albicans	99
NJ25-1	*erg24/ERG24* Candida albicans	100
NJ51-2	*erg24/erg24* Candida albicans	100
KPC1	*erg6/ERG6* Candida albicans	99
KPC8	*erg6/erg6* Candida albicans	99
ATCC 42720	Candida lusitaniae	101
CL130	*erg6* Candida lusitaniae	102

a Strains obtained from the 2015 and 2016 Madhani plates. Designated HM.#-xx for plate number (#) and well (xx).

The ergosterol supplementation and growth curve analysis were conducted in a 96-well plate. Strains were incubated overnight (∼18 h) at 30°C with 150-rpm shaking. Cells were then pelleted and resuspended in either pH 4 or pH 8 synthetic complete medium buffered with McIlvaine’s buffer (39). Resuspended strains were added to wells containing the same-pH synthetic complete medium with either 2 μg/ml or 0.02 μg/ml of ergosterol (Sigma)-Tween 80-ethanol (2-mg/ml stock as previously described in reference 82). Growth was then measured at an absorbance of 595 nm every 10 min for 42 h with shaking between readings and incubation at 30°C. Control wells containing vehicle alone (ethanol and Tween) were also measured in order to ensure that any growth rate change detected was due to the addition of ergosterol. One-way analysis of variance (ANOVA) and Dunnett’s multiple-comparison test were run on the last time point under each condition compared to the pH 8-alone condition to determine statistical significance. The pH of the medium in the wells was tested following the experiment to ensure that the medium remained buffered.

To generate the *sre1*Δ deletion and *eGFP-Rim101 *+* sre1*Δ deletion and tagged deletion constructs, respectively, we performed the previously described double-joint PCR with split drug resistance marker method to make targeted gene deletions (15, 83). In brief, we generated the following two PCR products: 5′ flanking region of the target locus (1,000 bp) with a truncated drug resistance cassette and the remainder of the drug resistance cassette with the 3′ flanking region of the target locus (1,000 bp). We then used biolistics to transform these two amplicons into either the wild-type C. neoformans strain (H99) or the C. neoformans strain that contains endogenously expressed GFP-Rim101 (84). Transformants were selected for the presence of the construct on YPD medium plus neomycin (NEO). To generate the fluorescently tagged *His-GFP-Sre1* strain, we used In-Fusion (Clontech) to clone the *SRE1* gene and terminator into the HGNAT (pCN19) plasmid, containing the GFP sequence and the nourseothricin (NAT) resistance marker (85). This plasmid was then biolistically transformed into the H99, wild-type (WT) strain. To generate the SRE1 reconstituted strain, we cloned the SRE1 gene and terminator into the pCH233 plasmid, containing the nourseothricin (NAT) resistance marker (86). This plasmid was then biolistically transformed into the *sre1*Δ (HEB5) strain. The primers used to generate each strain are listed in Table 2. Primers used to validate all *sre1*Δ mutants through Southern analysis (data not shown) are also listed in Table 2. Transformants were selected on YPD medium containing NAT (fluorescent strain) or NAT/NEO (reconstituted strain). Plasmids used in this study to amplify markers and clone new plasmids are listed in Table 3.

**TABLE 2:** Primers used in this study

Primer type and name	Primer sequence	Primer description^*a*^
Deletion constructs		
AA4950	AGGATTTGGGCAAATCGAGA	*SRE1* ko primer 1
AA4951	GTCATAGCTGTTTCCTGGGGAAAGAATCGTCTCATCA	*SRE1* ko primer 2
AA4952	ACTGGCCGTCGTTTTACAGGCGATGCTATCTATGGGT	*SRE1* ko primer 3
AA4953	GGAACCAATAAAGCGACCCA	*SRE1* ko primer 4
M13F	GTAAAACGACGGCCAGT	*NEO* cassette flank (F)
M13R	CAGGAAACAGCTATGAC	*NEO* cassette flank (R)
AA3935	CCTGAATGAACTGCAGGA	*NEO* internal cassette (R)
AA3934	TCGATGCGATGTTTCGCT	*NEO* internal cassette (F)

Reconstitution constructs		
AA5546	CGTCGCACTAGTGAGAGGGAGAAAGCTGGC	*SRE1* complement (F)
AA5547	CGTCGCACTTTTGGTGGACGGGCATTAATA	*SRE1* complement (R)

Southern probes		
AA4975	GGAACTGGCCAAATACGCAG	*SRE1* Southern probe (F)
AA4976	TTCCATGGTCCCTATCCATT	*SRE1* Southern probe (R)

Fluorescent constructs		
AA5514	GTACGGATCCACTAGTATGGCCTCATTACAGGACAAGATGC	*HIS-GFP-SRE1* (F) 1
AA5517	GGCGGCCGTTACTAGTACATCACGTACGTACATACAGC	*HIS-GFP-SRE1* (R) 2

a Abbreviations: ko, knockout; F, forward; R, reverse.

**TABLE 3:** Plasmids used in this study

Plasmid	Open reading frame	Backbone	Reference or source
pJAF	Neomycin (NEO) resistance cassette		103
pCN19	Histone H3 promoter; GFP	pJAF	85
pCH233	Nourseothricin (NAT) resistance cassette		86
pHEB13	Histone H3 promoter; GFP; *SRE1* including terminator	pCN19	This study

### Microscopy.

To analyze GFP-Rim101 localization in the WT, *rim20*Δ, and *sre1*Δ backgrounds, strains were incubated overnight (∼18 h) at 30°C with 150-rpm shaking. Cells were then pelleted and resuspended in either pH 4 or pH 8 synthetic complete medium buffered with McIlvaine’s buffer. Strains were shaken at 150 rpm and 30°C for 60 min as this has been shown to be sufficient time to observe the nuclear localization of Rim101 in WT cells (15). Fluorescent images were captured using a Zeiss Axio Imager A1 fluorescence microscope equipped with an Axio-Cam MRM digital camera. Images were created using ImageJ software (Fiji) (87).

### Protein extraction, immunoprecipitation, and Western blotting.

Protein extracts were prepared in a similar manner to what was previously described (15). Briefly, strains were incubated for ∼18 h at 30°C with 150-rpm shaking in YPD medium buffered to pH 4 or 5.5 with HEPES and HCl. Cells were then pelleted and resuspended in YPD medium buffered to pH 8 with HEPES and NaOH. These cells were incubated for 60 min and immediately pelleted and flash frozen on dry ice. Lysis was performed by bead beating (0.5 ml of 3-μm glass beads in a Mini-BeadBeater-16 [BioSpec] for 6 cycles of 30 s each with a 1-min ice incubation between bead-beating cycles for cell recovery). Supernatants were washed 3 times with 0.4 ml of lysis buffer (2× protease inhibitors [Complete, Mini, EDTA-free; Roche], 1× phosphatase inhibitors [PhosStop; Roche], and 1 mM phenylmethanesulfonyl fluoride [PMSF]). The crude pellet was pelleted through centrifugation at 15,000 rpm, 4°C, for 5 min, and the supernatant (cell lysate) was transferred (∼1 ml) to a new tube. For Western blotting assays assessing the presence of protein by probing for GFP, 50 μl of lysate was saved as whole lysate and 25 μl of GFP-trap (Chromotek) resin (equilibrated and resuspended in lysis buffer) was added to the remaining lysate. The lysates containing GFP-trap were incubated at 4°C for 2 h, rotating. Following incubation, lysates were spun down (2,500 × *g* for 2 min at 4°C) and washed 3 times with detergent-free buffer containing 2× protease inhibitors (Complete, Mini, EDTA-free; Roche), 1× phosphatase inhibitors (PhosStop; Roche), and 1 mM phenylmethanesulfonyl fluoride (PMSF). GFP-trap resin was resuspended in 4× NuPage lithium dodecyl sulfate (LDS) loading buffer and 10× NuPage reducing agent. Western blotting assays were performed using a 4% to 12% NuPage BisTris gel. To probe and detect GFP-Rim101 and GFP-Sre1, immunoblots were incubated with an anti-GFP primary antibody (using a 1/10,000 dilution; Roche), followed by a secondary anti-mouse peroxidase-conjugated secondary antibody (using a 1/25,000 dilution; Jackson Laboratory). Proteins were detected by enhanced chemiluminescence (ECL Prime Western blotting detection reagent; GE Healthcare).

For Western blotting assays assessing the presence of cleaved and uncleaved Sre1 using the polyclonal anti-Sre1, lysates were prepared in the same way as previously described. Following lysis and initial centrifugation of the crude pellet, 500 μl of lysates was precleared with 30 μl protein A-agarose (Sigma) and rotated for 1 h at 4°C. Lysates were incubated with 5 μl of anti-Sre1 polyclonal antibody (generously given to us by the Espenshade lab [21]) for 1 h. Protein A (60 μl/sample) was washed twice in lysis buffer and resuspended in equal volumes. Equilibrated protein A was then added to each lysate and incubated at 4°C for 1 h, rotating. Following incubation, lysates were spun down (2,500 × *g* for 2 min at 4°C) and washed twice with lysis buffer, once with lysis buffer plus 1 M NaCl, and twice with lysis buffer. Protein A resin was then resuspended in 4× NuPage lithium dodecyl sulfate (LDS) loading buffer and 10× NuPage reducing agent. Western blot assays were performed using a 3% to 8% NuPage Tris-acetate gel, with Tris-acetate running buffer. To probe for and detect Sre1, immunoblots were incubated in anti-Sre1 primary antibody (using a 1/200 dilution [21]) and then in anti-rabbit peroxidase-conjugated secondary antibody (using a 1/50,000 dilution; Jackson Laboratory). Proteins were detected in the same way as described above.

### Cell wall staining and flow cytometry.

For chitin and exposed chitin detection, cell wall staining with wheat germ agglutinin (WGA) and calcofluor white (CFW) was assessed as previously described (32). Briefly, overnight YPD cultures were diluted 1:10 in CO_2_-independent liquid medium and incubated (∼18 h) at 37°C with 150-rpm shaking. Cells were stained with 100 μg/ml of fluorescein isothiocyanate (FITC)-conjugated WGA and 25 μg/ml CFW and incubated in the dark for 35 min and 10 min, respectively. Quantitative analysis using ImageJ software was performed as previously described (32, 41).

For flow cytometry analysis, cells were incubated similarly as described above and fixed with 3.7% formaldehyde for 5 min at room temperature. Cells were then slowly pelleted and washed twice with phosphate-buffered saline (PBS). Cells were stained with 100 μg/ml FITC-conjugated wheat germ agglutinin (WGA; Molecular Probes). Cells stained with WGA were incubated in the dark at room temperature for 35 min. Cells were then slowly pelleted and washed twice with PBS. Cells from each strain were stained and resuspended in PBS at a concentration of 10^7^ cells/ml. Cells at 10^6^/ml were submitted to the Duke Cancer Institute Flow Cytometry Shared Resource for analysis using a BD FACSCanto II flow cytometer. Data were analyzed by FlowJo v10.6.1 software (FlowJo, LLC). Unstained cells were used as negative controls, and positive events were gated in the forward scatter/side scatterplots and represented as histograms. Geometric means were calculated based on the mean fluorescence intensity (*x* axis of histogram) of all cells quantified for each strain (*y* axis of histogram).

### Macrophage survival assay.

J774A.1 cells were incubated in a humidified 37°C incubator with 5% CO_2_, passaged twice weekly, and kept in tissue culture flasks in 20 to 25 ml of macrophage medium (Dulbecco’s modified Eagle’s medium [DMEM], heat-inactivated fetal bovine serum [FBS], penicillin-streptomycin [Gibco 15140-122], and minimal essential medium [MEM] nonessential amino acid solution [Gibco 11140-050]). Survival of C. neoformans strains within alveolar macrophage-like J744A.1 cells was assessed by aliquoting 100 μl of 10^5^ viable cells into a 96-well plate, avoiding edges as previously described (88). The plates were incubated overnight in a 37°C incubator with 5% CO_2_. Macrophages were then activated with 10 nM phorbol myristate acetate (PMA) and incubated at 37°C, 5% CO_2_, for 1 h. Fungal cells were incubated overnight (∼18 h) at 30°C with 150-rpm shaking. Cells were then pelleted, washed twice in PBS, and resuspended in macrophage medium. Fungal cells (10^6^ cells/ml) were opsonized with monoclonal antibody (Mab) 18B7 (1 μg/ml) for 1 h at 37°C. Cell concentrations were verified with quantitative culture. Macrophage medium was removed from the 96-well plate, and 100 μl of opsonized fungal cells was added to each well. The cocultures were incubated for 1 h at 37°C incubator with 5% CO_2_. Each well was then washed 3 times with PBS to remove extracellular yeast. One hundred microliters of macrophage medium was added to each well and incubated for 24 h at 37°C with 5% CO_2_. Following incubation, macrophage killing was determined by adding 200 μl sterile distilled water (dH_2_O) to each well, incubating at room temperature for 5 min, and assessing by quantitative cultures. One-way ANOVA and Tukey’s multiple-comparison tests were run to assess statistical significance between fungal cell survival percentages. Six biological replicates of each strain were analyzed.

### RNA-sequencing preparation and analyses.

WT and *sre1*Δ cells were incubated at 30°C with 150-rpm shaking in YPD medium to mid-logarithmic phase. Approximately 1 × 10^9^ cells from each strain were pelleted and resuspended in YPD medium buffered to pH 4 or pH 8 and incubated at 30°C for 90 min with 150-rpm shaking. All cells were pelleted, flash frozen on dry ice, and lyophilized overnight. This experiment was conducted with six biological replicates for the WT strain and the *sre1*Δ strain under both pH 4 and pH 8 conditions (24 samples total). RNA was isolated using the Qiagen RNeasy Plant minikit with optional on-column DNase digestion (Qiagen, Valencia, CA). RNA quantity and quality were measured using the Agilent 2100 Bioanalyzer. The NEBNext poly(A) mRNA magnetic isolation module was used to enrich for mRNA, and the NEBNext Ultra II directional RNA library prep kit for Illumina was used to prepare libraries (New England Biolabs, Ipswich, MA). Libraries were submitted to the Duke Sequencing and Genomic Technologies Shared Resource for sequencing on the Illumina NextSeq 500 with 75-bp, single-end reads.

Reads were mapped to the C. neoformans H99 reference genome (obtained from NCBI, accessed July 2019) using STAR alignment software (89). Differential expression analyses were performed in R using an RNA‐Seq Bioconductor workflow (90, 91) followed by the DESeq2 package with a false-discovery rate (FDR) of 5% (92). Genes were considered statistically differentially expressed if they had an adjusted *P* value of <0.05.

A modified Gene Ontology-term (GO-term) analysis using the FungiDB database was performed to identify genes that were significantly regulated in a given process as previously reported (15, 93). The differentially expressed genes in each category were determined based on two criteria: *P* value of <0.05 and base mean value of >20. Further differentiation was made based on the log_2_ fold change values. For the *sre1*Δ versus wild-type data set, we used a log_2_ fold change of ±1. For the positively regulated genes in the wild-type pH 4 versus pH 8 data set, we used a log_2_ fold change of 1, and for the negatively regulated genes in the wild-type data set, we used a log_2_ fold change of −3 due to the large amount of genes in this set. Fold change graphs were generated in GraphPad Prism (GraphPad Prism version 8.00 for Mac, GraphPad Software, San Diego, CA, USA), and Seaborn was used to visualize the DESeq2 results in a volcano plot (94). A complete list of the transcriptome sequencing (RNA-seq) data sets containing differentially expressed genes in each strain and associated with the appropriate GO-term category can be found in Table S1 in the supplemental material.

### Antifungal susceptibility tests.

For fluconazole and amphotericin B (AMB) Etest assays and pyrifenox disc diffusion, fungal cells were incubated overnight (∼18 h) at 30°C with 150-rpm shaking in YPD. Cells were normalized to an optical density at 600 nm (OD_600_) of 0.6 and diluted 1:10 in PBS, and 100 μl was plated to either YPD pH 5.5 or YPD pH 8 agarose plates. For the fluconazole and AMB Etest assay, an Etest strip (bioMérieux) containing a gradient of drug concentrations was placed on top of the plated fungal lawn. Plates were then incubated at 30°C for 72 (AMB) and 120 (fluconazole) h. Pyrifenox susceptibility was assessed by standard disc diffusion assays using 5 μl pyrifenox (Sigma-Aldrich; CAS number 88283-41-4; final concentration of 1.2 g/ml). Plates were then incubated at 30°C for 72 h. Zones of inhibition were determined as a surrogate of antifungal activity.

MIC testing of AMB against a pH gradient was performed by broth microdilution. AMB resuspended in dimethyl sulfoxide (DMSO) was serially diluted in synthetic complete medium buffered to pH 4, 5, 6, 7, or 8 with McIlvaine’s buffer in a 96-well plate with the highest concentration being 3.2 μg/ml. Fungal cells were incubated overnight (∼18 h) at 30°C with 150-rpm shaking in YPD. Cells were then normalized and diluted in synthetic complete medium buffered to pH 4, 5, 6, 7, or 8 with McIlvaine’s buffer and added to the corresponding pH well containing AMB. Plates were incubated at 30°C for 48 h, and the MIC was determined to be the lowest concentration of drug that led to no fungal cell growth.

### Data availability.

All raw and analyzed RNA-sequencing data have been submitted to the NCBI GEO database under accession no. GSE147109 (https://www.ncbi.nlm.nih.gov/geo/query/acc.cgi?acc=GSE147109).
