# Supplementary figures and images for: Sterol-Response Pathways Mediate Alkaline Survival in Diverse Fungi
Source: mBio. 2020 Jun 16;11(3):e00719-20. doi: 10.1128/mBio.00719-20 (PMC7298709; doi:10.1128/mBio.00719-20)

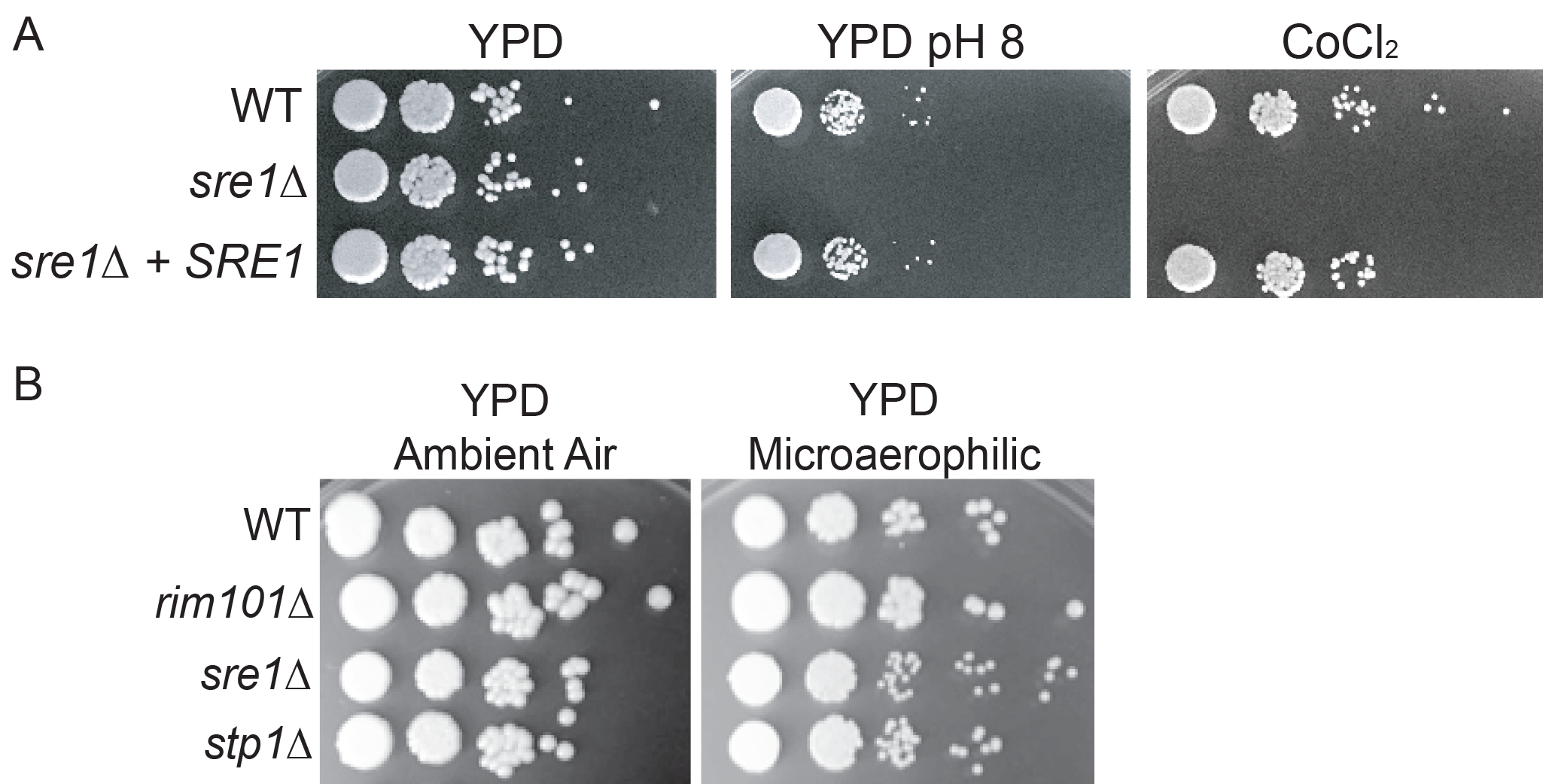

Supplement: FIG S1 [file mBio.00719-20-sf001.tif]

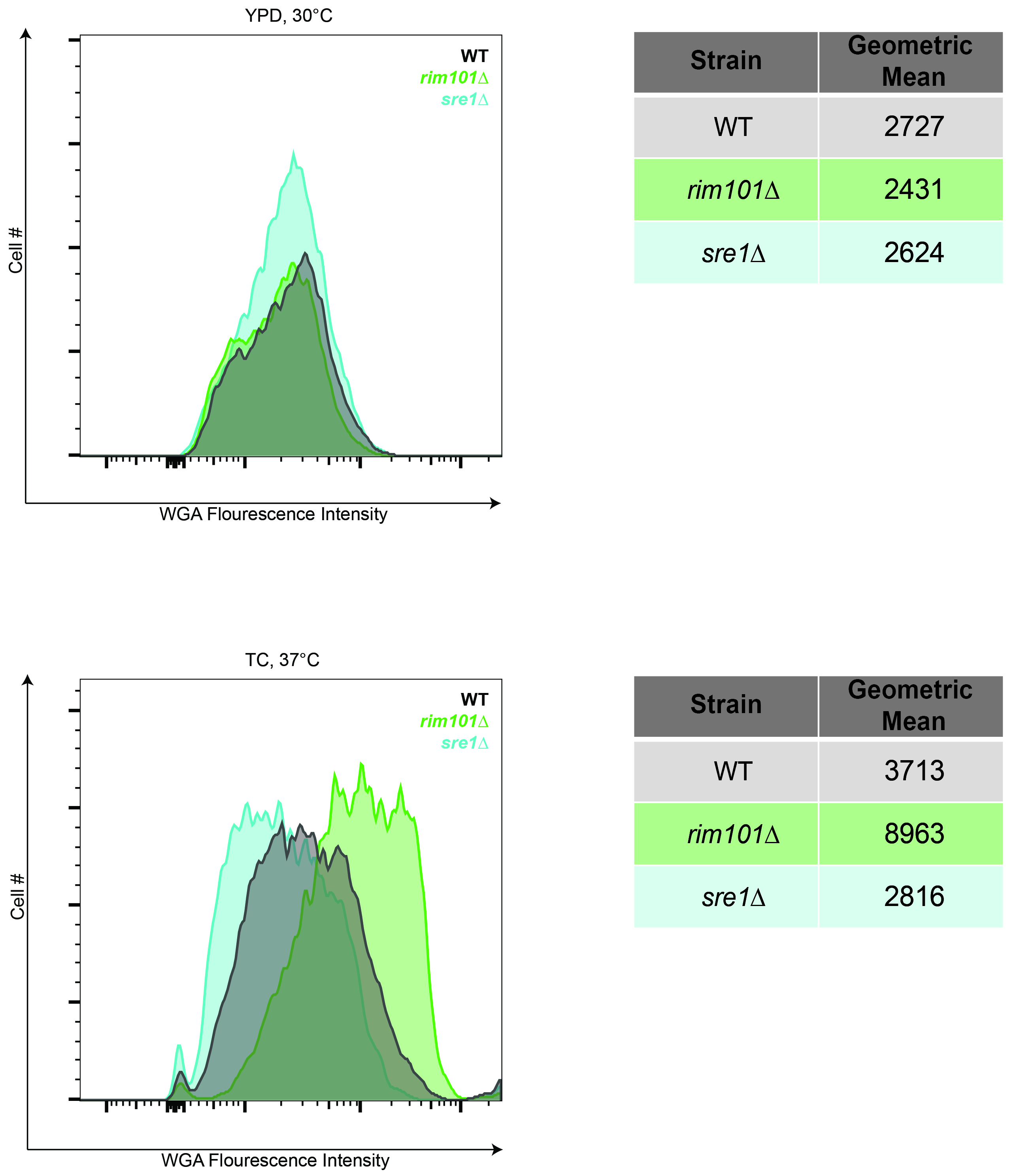

Supplement: FIG S2 [file mBio.00719-20-sf002.tif]

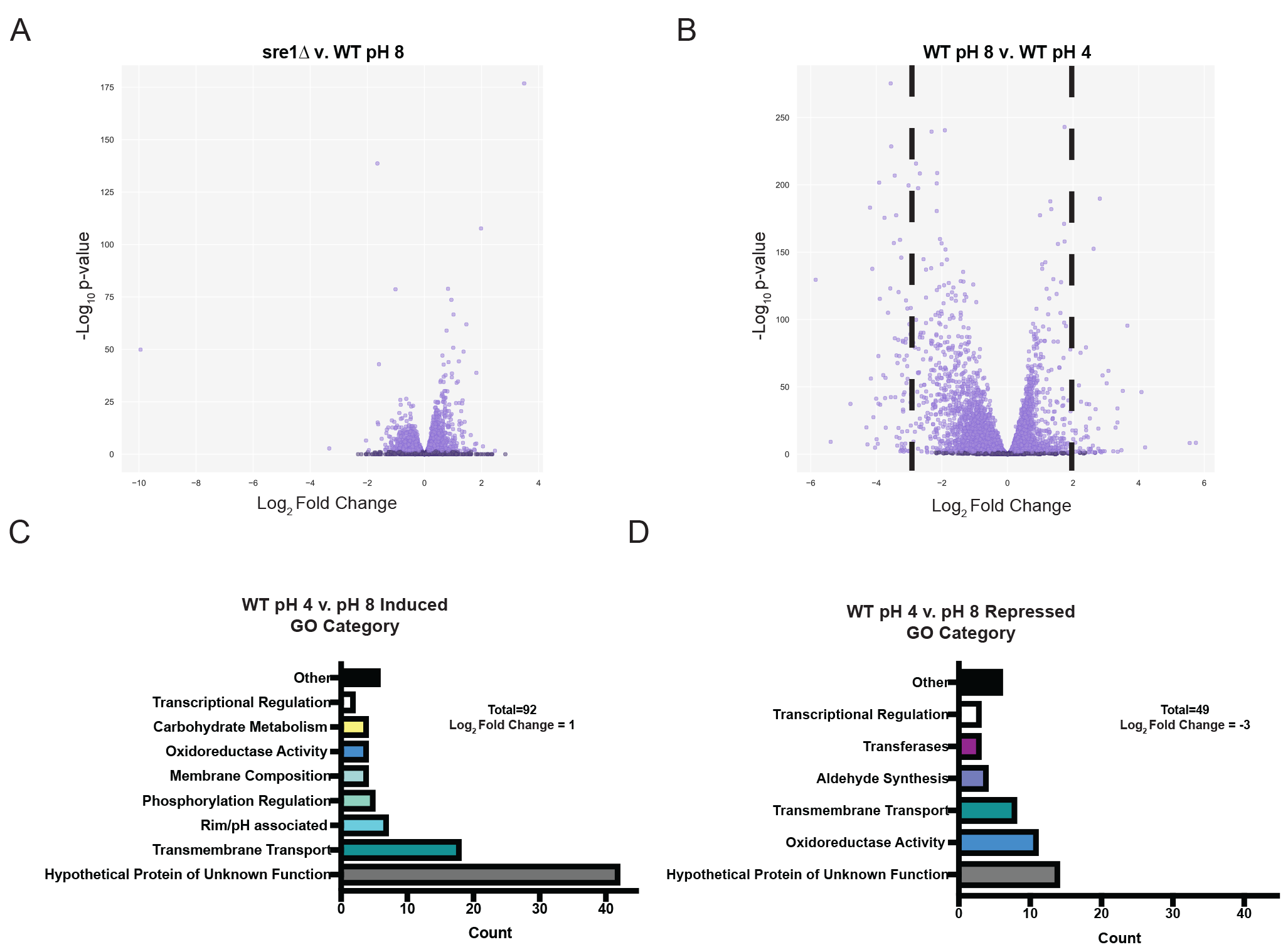

Supplement: FIG S3 [file mBio.00719-20-sf003.tif]

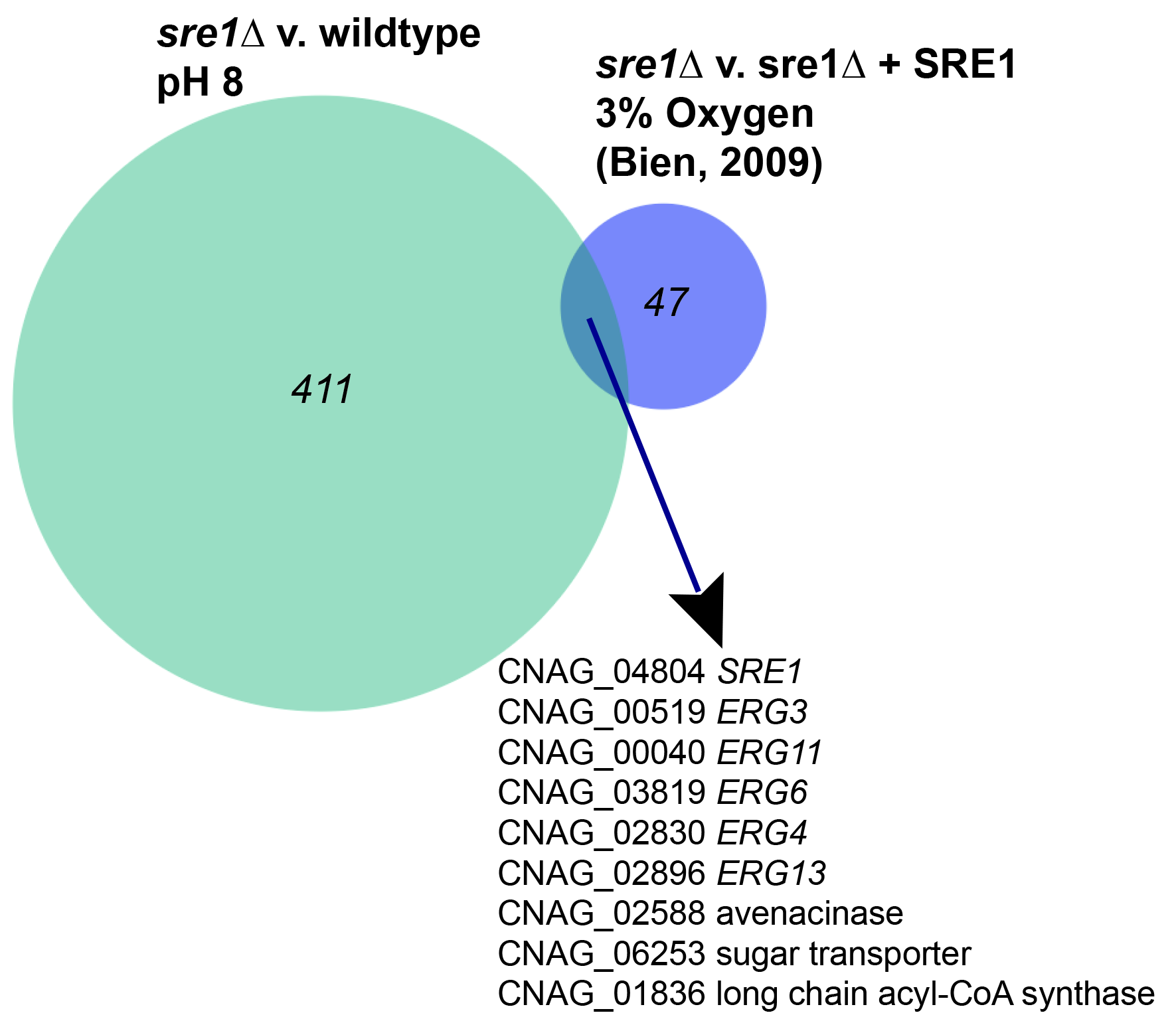

Supplement: FIG S4 [file mBio.00719-20-sf004.tif]
